# Methane Yield, Substrate Conversion, Microbial Community Structure and Metabolic Pathways During Anaerobic Digestion of Natural Cellulosic Biomass

**DOI:** 10.3390/bioengineering13060613

**Published:** 2026-05-25

**Authors:** Xiteng Chen, Hairong Yuan, Xiujin Li

**Affiliations:** Department of Environmental Science and Engineering, Beijing University of Chemical Technology, Beijing 100029, China; 2020400061@buct.edu.cn (X.C.);

**Keywords:** anaerobic digestion, natural cellulose, methane yield, microbial community structure, metabolic pathway

## Abstract

Three natural celluloses (softwood pulp, straw grass pulp, and degreased cotton) were used for anaerobic digestion tests to research methane yield, substrate conversion and microbial community structure, and further supplemented and clarified the metabolic pathway mechanisms of anaerobic digestion of cellulosic biomass. The results showed that natural cellulose could be significantly degraded and converted into methane by anaerobic microorganisms. The cumulative specific methane yields of wood pulp fiber (F1), straw pulp fiber (F2), and degreased cotton fiber (F3) were 373.57 ± 10.70 mL/g VS, 349.15 ± 13.20 mL/g VS and 346.16 ± 1.60 mL/g VS, respectively. The corresponding biodegradability values were 93.97%, 85.95% and 84.32%. Although the fermentation cycles in F1, F2, and F3 were identical (T95 was 12 days), the three groups exhibited distinct biogas production patterns. Metagenomic analysis indicated that F1 and F2 were dominated by the acetoclastic methanogenesis pathway, while the proportion of the hydrogenotrophic methanogenesis pathway increased in F3. Meanwhile, the cell motility pathway category was significantly enriched in F3. These results supplement the existing research on the anaerobic digestion of natural cellulose and provide theoretical support for the efficient anaerobic bioconversion of natural cellulosic biomass.

## 1. Introduction

Biomethane recovery through anaerobic digestion (AD) from lignocellulosic biomass is a promising approach to realize waste resource utilization, renewable energy recovery and carbon emission reduction [1]. Lignocellulosic biomass can be derived from various organic waste resources, including municipal solid waste (kitchen waste, garden trimmings, and paper products), agricultural wastes (crop straws, husks, and nut shells), and industrial organic waste (organic by-products from food processing, wood sawdust, and pulp residues) [2,3]. According to relevant reports, the output of crop straw in China reached 865 million tons in 2022. The global annual output of lignocellulosic biomass reaches 181.5 billion tons, while merely 8.2 billion tons are currently utilized [4,5]. Low-cost, high-yield and biodegradable lignocellulosic waste have become an extremely attractive resource for the biorefining industry [6]. Lignocellulosic biomass can be biologically converted through AD, which can generate bioenergy and alleviate the energy crisis [5].

Lignocellulosic biomass mainly consists of cellulose, hemicellulose, and lignin [7], Although considerable studies have explored the AD performance of individual fractions, most studies have mainly focused on regulating the operational parameters of anaerobic digestion, such as inoculation ratio, organic loading rate and temperature, while determining the biogas production and methane yield of different raw materials. Limited by sequencing technology and depth, most microbiological studies primarily focus on describing microbial community composition, while in-depth analyses of functional genes and metabolic pathways may remain relatively insufficient in most cases [8,9]. Cellulose and hemicellulose serve as the predominant carbon sources in anaerobic systems, which directly determine the efficiency of methane production from biomass [10]. As an aromatic polymer, lignin is not easily degraded by anaerobic microorganisms and contributes little to biogas generation in conventional AD processes [11]. Moreover, lignin can also wrap around polysaccharide components and further inhibit the biodegradation of cellulose and hemicellulose. Compared with hemicellulose, which has favorable biodegradability [12], cellulose exhibits higher crystallinity and a complex supramolecular structure, forming a dense physical barrier that significantly impedes the adsorption and binding of hydrolases, as well as the subsequent enzymatic degradation process. AD performance and the conversion efficiency of cellulose vary notably across existing studies. Such differences are closely associated with multiple key factors, including lignin content, cellulose crystallinity, and chemical modification [13].

Natural cellulose refers to a class of natural fiber feedstock derived from natural lignocellulosic biomass, with cellulose as its primary structural skeleton component. Multiple forms of natural cellulose, such as cotton, pulp fiber and microcrystalline cellulose, can all be regarded as the benchmark for biodegradability. However, most AD studies in laboratories adopt chemically purified cellulosic model substrates such as microcrystalline cellulose and α-cellulose to eliminate the interference caused by complex substrates [14,15,16]. However, although refined cellulose is chemically purified and has well-controlled substrate properties, its microscopic morphology and aggregation structure are significantly altered, and therefore it cannot fully represent the actual anaerobic digestion characteristics of natural cellulose. Industrial and agricultural natural cellulose resources, including pulp fibers and cotton cellulose, are mostly applied as auxiliary substrates in co-digestion systems [17,18]. Few studies have investigated multiple representative types of natural cellulose, and limited attention has been given to the anaerobic fermentation gas production performance and methane potential comparison of three natural cellulose substrates (softwood pulp fiber, straw pulp fiber, and degreased cotton fiber). Meanwhile, the microecological mechanism during natural cellulose anaerobic digestion remains poorly understood [19]. Furthermore, prominent research gaps still exist in the assembly patterns of functional microbiomes, taxonomic identification of core microbial consortia, and metagenome-based regulation of metabolic pathways, material conversion and energy allocation.

This study investigated the differences in methane production performance among three natural cellulose substrates. By systematically elucidating their anaerobic digestion characteristics, this work complemented existing studies on the anaerobic digestion of natural cellulose substrates and further clarified the substrate-dependent differences in methane production potential and substance conversion. Specifically, the main objectives of this study were: (1) to investigate the methane production potential, biogas production dynamics, methanogenic kinetics and cellulose conversion efficiency of different natural cellulose materials; (2) to clarify the community structure and microbial co-occurrence network of core functional microbial consortia during the anaerobic degradation of natural cellulose; and (3) to characterize the key metabolic pathways involved in cellulose decomposition and methanogenesis at the metagenomic level and further reveal the differential metabolic regulation mechanisms among natural celluloses with different structural features.

## 2. Materials and Methods

### 2.1. Feedstocks and Inoculum

**Figure 1:**
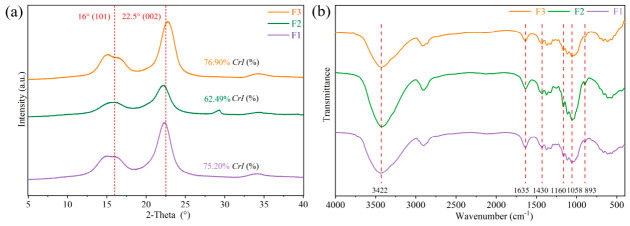
XRD (**a**) and FT-IR (**b**) of three natural celluloses.

Natural cellulose mainly includes pulp fiber, cotton cellulose, microcrystalline cellulose, and other highly pure cellulose-based feedstocks. Representative feedstocks (softwood pulp, straw grass pulp, and degreased cotton) were selected for AD respectively, as shown in Figure S1. The softwood pulp fiber (F1) employed in the experiments was obtained from sulfate-processed pulp boards made from coniferous species. The straw grass pulp fiber (F2) was derived from sulfate-processed pulp boards produced using wheat straw and corn stover [20]. The degreased cotton fiber (F3) was sourced from common experimental materials (Shandong Huachen, Weifang, China). Softwood pulp fibers, straw fibers, and degreased cotton fibers were subjected to mechanical crushing pretreatment prior to the experiment. After treatment, the materials became loose and fluffy, allowing them to mix thoroughly in the anaerobic system and achieve as uniform a distribution as possible. Softwood pulp underwent a bleaching process, while straw grass pulp remained unbleached. Water-soluble sulfates and chlorides in the samples were tested in accordance with the methods described in the national standards of the People’s Republic of China (GB/T 2678.6-2023 and GB/T 22904-2023) [21,22], to avoid unpredictable impacts on anaerobic microorganisms. It has been found in our previous work that anaerobic granular sludge exhibits favorable methane production potential [12]. Anaerobic granular sludge was collected from the anaerobic unit of the secondary treatment process in a wastewater treatment plant located in Weifang City, Shandong Province, China. Anaerobic granular sludge served as the inoculum for the experiments, which was degassed before application in the tests [12]. Characteristics of inoculum and feedstocks are shown in Table 1. The crystalline structural characteristics of natural cellulose fibers were characterized via X-ray diffraction (XRD), and the chemical composition and functional group distribution were analyzed by Fourier transform infrared spectroscopy (FT-IR) (Figure 1).

### 2.2. Batch Anaerobic Digestion

Batch anaerobic digestion experiments were conducted using AMPTS II tester (BPC Instruments, Jiaxing, China). The fermentation bottles were operated under mesophilic conditions (35 ± 0.5 °C) with a working volume of 400 mL. The reactors were operated at an organic loading rate of 20 g VS/L, with an inoculum-to-substrate ratio (ISR, based on VS) of 2 and a carbon-to-nitrogen ratio (C/N) of 25:1 (based on previous research work) [12]. Tap water was added to adjust the initial system total solid (TS) content to approximately 8%. Before the beginning of the experiment, the initial pH was set at 7.5. The hydraulic retention time for biochemical methane potential (BMP) test was set at 25 days, with mechanical stirring applied at a rate of 100 rpm for 5 min per hour. All assays were performed in triplicate, with a blank control containing only inoculum sludge to account for background biogas production. Daily biogas production and composition were monitored throughout the digestion period, and gas volumes were normalized to standard temperature and pressure (273.15 K, 101.325 kPa). At the end of anaerobic digestion, the total solids (TS), volatile solids (VS), and cellulose content in each experimental group were measured to calculate their respective conversion efficiencies.

### 2.3. Analytical Methods

#### 2.3.1. Physical and Chemical Properties

Biogas compositions including CH_4_ and CO_2_ were analyzed by gas chromatography (GC-2014c, Shimadzu, Kyoto, Japan). The instrument was equipped with a TDX-01 column and a thermal conductivity detector (TCD). High-purity argon (99.999%) served as the carrier gas at a constant flow rate of 30 mL/min. The content of individual biogas components was quantified using the external standard method, and all results were presented as volume percentage (% *v*/*v*). The total solids (TS), volatile solids (VS) and pH value were measured through standard methods (APHA2012) [23]. Cellulose content was analyzed using a fully automatic cellulose analyzer (F2000, Hanon, Jinan, China). Prior to cellulose content determination, all tested samples underwent homogenization, were dried at 105 °C to a constant weight, and subsequently crushed, ground, and sieved through a 10-mesh screen. The mass of the sample added to each filter bag ranged from 0.5 g to 1 g. Ethanol and volatile fatty acids (VFAs) were analyzed using gas chromatography with high-purity nitrogen as the carrier gas (GC-2014c, Shimadzu, Kyoto, Japan). Ammonia nitrogen was measured by an environmental testing photometer (HI83206, HANNA instruments, Woonsocket, RI, USA) equipped with the HI93700-01 ammonia low range reagent kit. The morphological characteristics of natural cellulose fibers were examined by scanning electron microscopy (SEM, S-4700, Hitachi, Japan). The crystalline structure of natural cellulose fibers was characterized using X-ray diffraction (XRD, D/max-2200PC, Rigaku, Tokyo, Japan), while the chemical composition and functional group distribution were analyzed by Fourier transform infrared spectroscopy (FT-IR, Nicolet iS20, Thermo Fisher Scientific, Waltham, MA, USA).

#### 2.3.2. Kinetic Equations

The modified Gompertz, logistic, cone, and first-order kinetic model equations were used to fit the cumulative specific methane yield, as shown in Equations (1)–(4). *B* refers to cumulative specific methane production (mL/g VS). *B*_0_ denotes the maximum potential methane yield (mL/g VS). *R*_max_ represents the highest daily methane production rate (mL/g VS/d). Additionally, *λ* indicates the lag phase duration (d), *t* is the fermentation reaction time (d), and *k* stands for the reaction rate constant (d^−1^). e is a mathematical constant with an approximate value of 2.7183. According to the elemental composition of three natural cellulose, the theoretical cumulative methane yield (TMY) was calculated through the total biochemical reaction equation of anaerobic digestion, as shown in Equations (5) and (6). The theoretical specific methane yields of softwood pulp fiber, straw pulp fiber and degreased cotton fiber are 397.56 mL/g VS, 406.23 mL/g VS and 410.54 mL/g VS, respectively. The biodegradability (BD) of the raw materials was calculated in Equation (7). EMY in Equation (7) refers to the experimental cumulative methane yield.

(1) $$B=B_{0}\times \exp\left\{-\exp\left[\frac{R_{max}\times e}{B_{0}}\left(\lambda -t\right)+1\right]\right\}$$

(2) $$B=\frac{B_{0}}{1+exp\left[\frac{{4R}_{max}}{B_{0}}\left(\lambda -t\right)+2\right]}$$

(3) $$B=\frac{B_{0}}{1+{\left(kt\right)}^{-n}}$$

(4) $$B=B_{0}\times \left[1-\exp\left(-kt\right)\right]$$

(5) $$\begin{array}{c} C_{n}H_{a}O_{b}N_{c}+\left(n-\frac{a}{4}-\frac{b}{2}+\frac{3c}{4}\right)H_{2}O\to \left(\frac{n}{2}+\frac{a}{8}-\frac{b}{4}-\frac{3c}{8}\right){CH}_{4}+\\ \left(\frac{n}{2}-\frac{a}{8}+\frac{b}{4}+\frac{3c}{8}\right){CO}_{2}+c{NH}_{3} \end{array}$$

(6) $$TMY=\frac{22.4\times 1000\times \left(\frac{n}{2}+\frac{a}{8}-\frac{b}{4}-\frac{3c}{8}\right)}{12n+a+16b+14c}$$

(7) $$BD=\frac{EMY}{TMY}\times 100\%$$

### 2.4. Metagenomic Analysis

The DNA extraction of samples was carried out using the E.Z.N.A.^®^ soil DNA Kit (Omega Bio-tek, Norcross, GA, USA) according to manufacturer’s protocols. DNA extract was fragmented to an average size of about 350 bp using Covaris M220 (Gene Company Limited, Hong Kong, China) for paired-end library construction. Paired-end library was constructed using NEXTFLEX^®^ Rapid DNA-Seq (Bioo Scientific, Austin, TX, USA). Paired-end sequencing was performed on Illumina NovaSeq™ X Plus (Illumina Inc., San Diego, CA, USA) at Majorbio Bio-Pharm Technology Co., Ltd. (Shanghai, China) using the NovaSeq X Series 25B Reagent Kit according to the manufacturer’s instructions. The data were analyzed on the free online platform of Majorbio Cloud Platform (www.majorbio.com) [24]. The quality-filtered data were assembled using MEGAHIT (https://github.com/voutcn/megahit, version 1.1.2, accessed on 20 December 2025) to remove low quality reads, splice sequences and host contamination. Taxonomic and functional annotation analyses of the obtained gene sequences were performed by aligning against the National Center for Biotechnology Information (NCBI) and Kyoto Encyclopedia of Genes and Genomes (KEGG) databases.

### 2.5. Statistical Analysis

Data recording and statistics were performed using Microsoft Excel 2021. Kinetic fitting and plotting were performed with Origin 2026. FT-IR data were analyzed using OMNIC 9.2.86 (Thermo Fisher Scientific, Waltham, MA, USA). Venn diagrams and Circos plots were generated using Majorbio Cloud Platform (https://www.majorbio.com/tools, accessed on 1 March 2026). Gephi 0.9.1 was used for microbial bipartite network visualization. Raw functional gene abundance was Z-score normalized in R for dimensionality unification. A multiple group heatmap of batch matrices was generated on the CNSKnowall platform (https://www.cnsknowall.com, accessed on 15 March 2026).

## 3. Results and Discussion

### 3.1. Methane Production Performance

**Figure 2:**
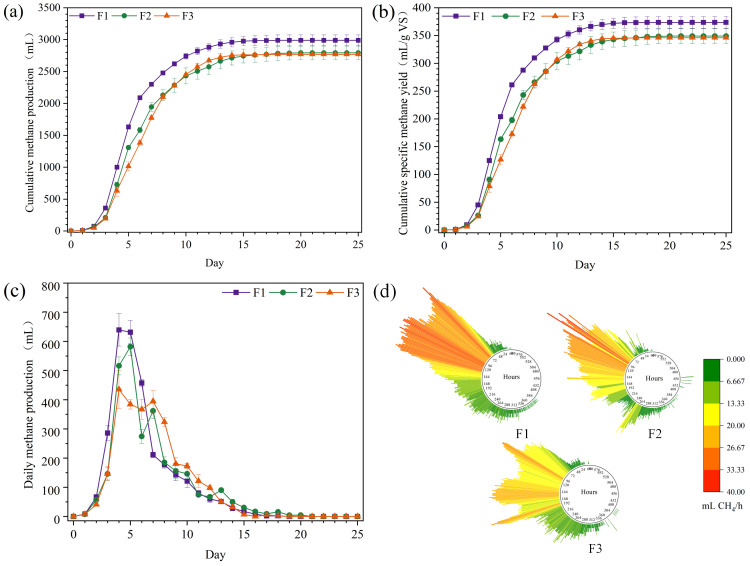
Cumulative methane production (**a**), cumulative specified methane yield (**b**), daily methane production (**c**), and methane production rate per hour (**d**) from different groups.

The cumulative biogas production trends of the three natural cellulose substrates were generally consistent, although noticeable differences existed among groups. As shown in Figure 2, after a short microbial acclimation period (approximately 1~2 days), all three substrates rapidly entered the logarithmic gas production stage and achieved their methane production peaks, which predominantly occurred on the fourth day. A similar trend was observed for pure cellulose, implying that natural cellulosic substrates could be efficiently hydrolyzed and acidified. Daily methane production declined rapidly after the 5th day, and remained at a low and stable level starting on the 12th day (Figure 2c). Readily degradable organic fractions were gradually depleted, and the anaerobic digestion process gradually approached completion.

The theoretical specific methane yield of α-cellulose (pure cellulose substance) was around 438 mL/g VS [25]. Despite failing to reach the theoretical specific methane yield of pure cellulose, the three natural cellulose substrates exhibited considerable methane production performance. In terms of cumulative methane production, Group F1 exhibited optimal biogas production potential, with its cumulative specific methane yield (Figure 2b) reaching 373.57 ± 10.70 mL/g VS, which was significantly superior to that of Groups F2 and F3 (349.15 ± 13.20 mL/g VS and 346.16 ± 1.60 mL/g VS). The theoretical specific methane yields of wood pulp fiber (F1), straw pulp fiber (F2) and degreased cotton fiber (F3) were 397.56 mL/g VS, 406.23 mL/g VS and 410.54 mL/g VS, respectively. The above result indicates that the biodegradability (BD) in Group F1 reaches 93.97%, which is much higher than 85.95% and 84.32% achieved by Groups F2 and F3. The methane yield and BD value of the three experimental groups were close to or even higher than those of the previous studies [19,26], which proved that natural cellulose had the potential to be efficiently converted into methane.

The AD performance of the three substrates was also reflected in the daily biogas production and cumulative curves (Figure S2a,b). Group F1 rapidly climbed to a peak of approximately 1446.66 ± 136.71 mL/d on the 4th day of AD, and the cumulative biogas production of F1 was the highest (5854.76 ± 99.76 mL), which was 14.35% and 10.49% higher than that of F2 and F3, respectively. In comparison, the peak value of Group F2 was 1179.57 ± 47.18 mL/d, while that of Group F3 was only 908.24 ± 20.20 mL/d. During anaerobic digestion, methane content increased gradually, while CO_2_ content followed an initial rise and subsequent continuous decline in all groups (Figure S2c,d). Group F1 exhibited a pronounced early methanogenic burst (Figure 2d), with methane content rapidly rising and stabilizing at approximately 65%. During the stable period of late fermentation, F2 maintained the highest methane content among all groups, along with the fastest declining rate of carbon dioxide. In contrast, the methanation rate of F3 was generally lower, and the peak gas production was more dispersed. Specifically, during the 8th to 15th day of fermentation, the methane content decreased significantly, while the carbon dioxide concentration maintained a high level of 35% to 40% for a long period. Cumulative biogas production of F3 was slightly higher than that of F2. This difference may be attributed to the sustained release of high-concentration CO_2_ during the mid-to-late anaerobic digestion stage, along with continuous gas production potentially derived from the slow degradation of cellulosic components. The limited cumulative biogas production of F2 could be attributed to its lowest cellulose content (Table 1), which resulted in insufficient carbon sources and weakened biodegradation capacity in the later stage.

F1, F2 and F3 demonstrated three different biogas generations (Figure 2d). F1 exhibited a higher biogas yield during the initial fermentation stage, a shorter digestion period, and a sustained increase in methane content, indicating the dynamic balance between acidogenesis and methanogenesis. Despite F2 exhibiting a comparable biogas production peak to F1, its gas production intensity was relatively lower. F2 presented a relatively earlier methanogenic startup and a faster increase in methane concentration. It may be attributed to the abundant readily biodegradable components, especially hemicellulose, in its substrate. F3 showed delayed initial gas production, supported by sustained decomposition of high-purity cellulose, followed a slow, long-term fermentation mode with continuous and stable biodegradation performance. Based on the SEM and XRD results (Figure 1a and Figure S1), degreased cotton fiber was found to possess a dense, smooth, and compact structure with relatively high crystallinity. It is possible that these properties partially contributed to the observed difference in methane production compared with pulp fibers.

### 3.2. Kinetics Analysis of Methane Yield

The time taken to reach 95% of the maximum methane yield (T95) was 12 days for F1, F2 and F3. The T50 (time to 50% maximum biogas production) of F1 was only 5 days, which was 1 day earlier than that of F2 and F3 (both 6 days). The T50 and T90 times of paper waste with similar compositional characteristics were 13 days and 33 days respectively in a previous study [19]. The results indicated that natural cellulose could be effectively degraded during anaerobic digestion, the inoculum exhibited high biological activity, and the anaerobic reactor maintained stable operation throughout the experiment. The curve fitting of four models are shown in Figure S3 [27]. Modified Gompertz and cone models could describe the experimental process with a high coefficient (R^2^ > 99.50%) [28]. The fit coefficient of the first-order kinetics model was the lowest, indicating that the reaction process was not a simple first-order reaction but rather involved a significant lag phase or complex degradation stage. The excellent fitting performance of the modified Gompertz model was consistent with the previous related studies [14,29]. The maximum methane production potential (*B*_0_), the highest maximum methane production rate (*R*_max_), and the shortest lag phase (*λ*) were achieved by the F1 group. It was indicated by these results that the microbial metabolic activity and performance in the F1 group were superior to those in the other two groups. In Group F1, the shortest lag phase time was 2.24 d. While the largest lag phase time was 2.72 d in F3 (Table 2). A relatively slow degradation rate was observed in F3, which was considered to indicate a slow-release and long-lasting fermentation characteristic.

Only the *B*_0_ value (modified Gompertz model) of F1 was close to the results of microcrystalline cellulose reported in the literature [14,15], but the *λ* values of F1, F2 and F3 were lower than those in the previous studies, and the lag phase was within 3 days for all groups [16]. These results indicated that the inoculum used in this study exhibited better activity and a higher degradation capacity for cellulose. The *B*_0_ value in the cone model of F1 reached 375.61 mL/g VS, which was significantly higher than that of F2 and F3. Meanwhile, the highest reaction rate constant (*k*) of 0.20 d^−1^ was observed in F1, followed by F2 (0.18 d^−1^) and F3 (0.17 d^−1^). It was demonstrated by the results that optimal biodegradability and overall AD efficiency were achieved in F1, whereas the characteristics of a slower degradation rate and a more persistent degradation process were exhibited by F3. Furthermore, this variation pattern was found to be consistent with the biogas production of the three substrates.

### 3.3. Substrate Conversion

**Figure 3:**
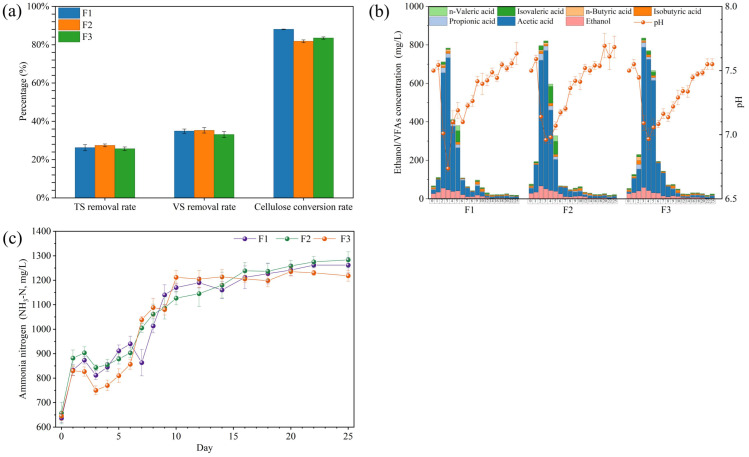
TS/VS removal rate and cellulose conversion rate (**a**); ethanol/VFA concentration and pH value (**b**); ammonia nitrogen (**c**). The cellulose conversion rate was calculated based on TS. The concentrations of ethanol, volatile fatty acids (VFAs), pH value and ammonia nitrogen were determined at anaerobic digestion days 0, 1, 2, 3, 4, 5, 6, 7, 8, 9, 10, 12, 14, 16, 18, 20, 22 and 25.

As illustrated in Figure 3a, three natural cellulose exhibited higher volatile solid (VS) removal rates (approximately 35%) than total solid (TS) removal rates (approximately 26%) after AD. In the FTIR spectra (Figure 1b), all three samples displayed typical absorption peaks of cellulose, without any significant characteristic signals of hemicellulose (C=O) and lignin (aromatic skeletal vibration) near 1730 cm^−1^ and 1510 cm^−1^. These results demonstrated that cellulose was the predominant chemical component in F1, F2, and F3. Cellulose conversion rates between 82% and 88% were observed across all reactor groups, with the highest conversion efficiency being achieved by F1. It was indicated by this remarkable efficiency that the hydrolysis and acidification of cellulose-the typically rate-limiting steps in AD-were highly effective in this study. Anaerobic microbial community had a significant metabolic capacity for the breakdown and bioconversion of complex lignocellulosic structures into methane. Wide variations in cellulose degradation performance have been found in anaerobic reactors, ranging from 30% to 95% [30,31]. Hubbe et al. found that some studies had demonstrated that cellulose could achieve nearly 100% biodegradation [13,32]. However, the hydrolysis conversion rate of cellulose gradually declined over time in most studies focusing on the kinetics of cellulose biodegradation, and ultimately, complete degradation and conversion of cellulose could not be achieved. This incomplete bioconversion could be attributed to the generation of inhibitors, non-productive adsorption onto lignin, surfactant effects, nutrient starvation within confined reactors, and the deposition of non-hydrolyzable substances [13].

As shown in Figure 3b, the pH values of F1 and F2 decreased rapidly from approximately 7.5 to below 7.0 on day 2, whereas the pH of F3 remained relatively stable at around 7.4 during the initial stage. The substrates (pulp fibers) were more susceptible to hydrolysis and acidification than degreased cotton fiber. Acetic acid dominated the VFA profile during the acidification phase in all three groups, indicating that the anaerobic fermentation of natural cellulose primarily followed the acetic-acid-type pathway. On day 3, the pH values in the F1 and F2 reactors reached their lowest levels, coinciding with the peak concentrations of total volatile fatty acids (TVFAs). Notably, VFA accumulation in F3 was the most pronounced, with elevated concentrations persisting for a longer period, as reflected by the high acetic acid levels maintained on days 5 and 6. Subsequently, as methanogens consumed the accumulated VFAs, the concentrations of all intermediate metabolites rapidly declined to low levels. On the 12th day, the concentrations of acetic acid and TVFA had already decreased to very low levels and remained at similarly low levels until day 25. Meanwhile, the pH values recovered to the optimal range for methanogens (7.3~7.7). The ammonia nitrogen concentrations in all three groups increased gradually during the initial stage of anaerobic digestion and stabilized at 1200~1300 mg/L after the 16th day (Figure 3c). Notably, these levels remained well below the reported inhibitory threshold for methanogens, indicating that ammonia inhibition was not a concern in any of the reactors.

### 3.4. Microbial Community Composition and Diversity

**Figure 4:**
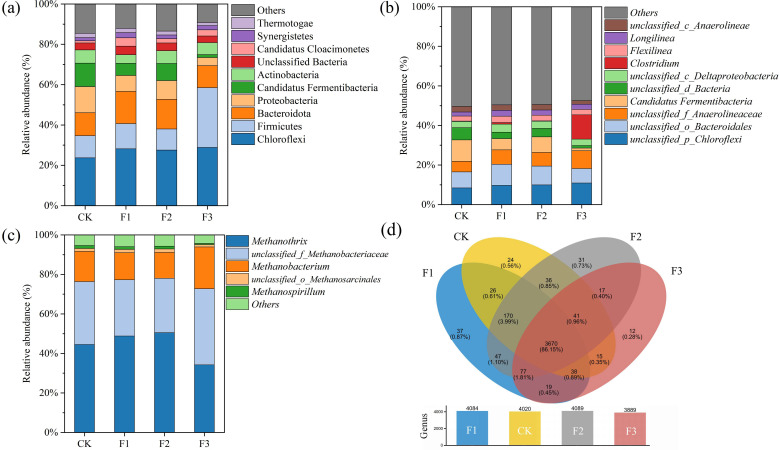
Microbial community composition in different anaerobic digestion groups. Bacterial community composition at the phylum level (**a**); bacterial community composition at the genus level (**b**); archaeal community composition at the genus level (**c**); Venn diagram of shared and unique genera, and the total number of genera in each group (**d**).

Anaerobic digestion stability and efficiency were fundamentally driven by synergistic interactions among the microbial community. Samples for metagenomic sequencing were collected on the 12th day to investigate the microbial community structure across various natural cellulose experimental groups. At this time point, the AD system had reached a stable state, and the microbial community structure tended to be stable, which could avoid the interference of unstable microbial community changes during the peak production stage on the analysis of the core functional microorganisms associated with substrate degradation. Previous studies have widely reported that Chloroflexi, Firmicutes (syn. Bacillota) and Bacteroidota are universally dominant phyla in anaerobic digestion systems fed with cellulosic biomass, which play core roles in substrate hydrolysis and acidification [9,14]. At the phylum taxonomic level, Chloroflexi, Firmicutes, and Bacteroidota, and Proteobacteria were the dominant phyla in all three groups. As shown in Figure 4a, Chloroflexi dominated the CK group (anaerobic granular sludge) with the highest relative abundance of 23.69%, followed by Proteobacteria (12.94%), Candidatus_Fermentibacteria (11.68%), Bacteroidota (11.36%), and Bacillota (10.97%). In the F1 group, Chloroflexi accounted for 28.13% and Bacteroidota for 15.92%, while Firmicutes reached 12.53% in relative abundance. The relative abundances of Chloroflexi, Bacteroidota, and Firmicutes in the F2 group were 27.56%, 14.73%, and 10.43% respectively, and the microbial community structure of F2 was highly similar to that of the F1 group. F3 was mainly dominated by Firmicutes (29.62%) with hydrolytic fermentation potential, while the relative abundances of Chloroflexi and Bacteroidota were 28.84% and 10.94%, respectively. Firmicutes represent a key hydrolytic phylum in the hydrolysis and acidogenesis stages of AD, which contains a large number of cellulolytic microorganisms capable of secreting various extracellular degrading enzymes [33]. Compared with pulp fiber, the cellulose structure of degreased cotton fiber was denser, more stable and had stronger resistance to degradation. The structural difference might lead to the substantial enrichment of Firmicutes in the F3 system to meet the demand for degrading recalcitrant cellulose, while relevant reports noted that the relative abundance of Firmicutes in the control group was higher than that in the pretreatment groups with disrupted lignocellulosic structures [34,35].

In F3, the relative abundance of *Clostridium* (phylum Firmicutes) increased significantly, and Group F3 was the only one in which the abundance of this genus exhibited an upward trend (Figure 4b). *Clostridium* is a key anaerobic hydrolytic bacterium frequently enriched in lignocellulosic AD systems. It encodes cellulolytic and hemicellulolytic enzymes, contributing to the hydrolysis of cellulose and hemicellulose and the production of volatile fatty acids. In environments rich in cellulose and hemicellulose, the genus Clostridium was enriched to a certain extent [10]. Song et al. also found that *Clostridium* was the crucial bacteria that facilitated the biodegradation of paper waste during AD [36]. The relative abundance of *unclassified Bacteroidales* (phylum Bacteroidota) in the F1 group exceeded that in the CK and F3 groups. The diverse hydrolytic bacterial community structure formed by the coexistence of Bacteroidota and other hydrolytic taxa may have contributed to maintaining the metabolic stability of the F1 system [37]. The genus-level archaea of F1 and F2 were predominantly dominated by *Methanothrix*, a typical acetoclastic methanogen with high acetate utilization efficiency [38]. Compared with the control, the relative abundance of *Methanothrix* declined in F3, whereas the family Methanobacteriaceae and genus *Methanobacterium* (hydrogenotrophic methanogens expanded significantly (Figure 4c). Such rearrangement of the methanogenic community indicated the presence of a potential metabolic limitation in the acetoclastic methanogenic pathway in F3, which might have favored a compensatory shift towards hydrogenotrophic methanogenesis. This microbial functional transition may have contributed to the prolonged lag phase (*λ*) and lower maximum methane production rate (*R*_max_) observed in the F3 group.

As shown in the metagenomic Venn diagram (Figure 4d), despite the variations in methanogenic performance, all reactors shared a substantial core microbiome comprising 3670 genera (representing 86.15% of the total annotated taxa). This high taxonomic overlap indicated that the inoculum sludge possessed a highly diverse microbial consortium with relative functional redundancy [39]. Although varied substrates had reshaped the relative abundance of specific key microbes, the overall taxonomic composition of the core community remained relatively stable, indicating that the microbes in anaerobic granular sludge could adapt to natural cellulosic biomass without the need for prolonged acclimation. In addition, it further indicated that natural cellulose possessed high biodegradability and could be efficiently degraded by the anaerobic microbial consortia. Based on metagenomic analysis, methanogenic archaea occupied an absolute dominant position in the three natural cellulose anaerobic digestion systems, which was clearly visualized in the Circos plots (Figure S4) and the bipartite network diagram (Figure S5). In this study, three of the top five most abundant genera across the entire microbial community were *Methanothrix*, *unclassified_f_Methanobacteriaceae*, and *Methanobacterium*. Unlike conventional systems that required sustained bacterial activity to break down recalcitrant organic matter, the natural cellulose was rapidly and efficiently degraded. In the late phase of anaerobic digestion, the depletion of readily biodegradable carbon substrates resulted in a reduced relative abundance of acidogenic populations. Meanwhile, the stable operational environment, characterized by low volatile fatty acid levels and minimal inhibition, likely provided a favorable niche for methanogenic archaea [10,40].

### 3.5. Metagenomic Analysis of Metabolic Characteristics

At KEGG level 1, metabolism was the predominant functional category, exhibiting the highest abundance with approximately 1.0 × 10^7^ reads (Figure S6a,c). Across the control (CK) and treatment groups (F1, F2, F3), metabolic functions consistently accounted for over 70% of the total annotated reads, followed by genetic information processing and environmental information processing. At KEGG level 2, carbohydrate metabolism, amino acid metabolism, energy metabolism, and the metabolism of cofactors and vitamins were major metabolic sub-categories [41]. Notably, the F2 group exhibited the highest absolute abundance in both carbohydrate metabolism and amino acid metabolism (Figure S6b). The elevated genetic potential for these metabolic pathways aligned with high microbial richness and provided a solid functional basis for the rapid reactor startup and efficient substrate degradation detected in the F2 group. The F3 group exhibited remarkable enrichment in cell motility within the cellular processes category (bacterial chemotaxis and flagellar assembly pathways in KEGG level 3) (Figure S6b,d). The enrichment of genes associated with cell motility has been suggested to potentially promote the availability of carbon substrates and methane production [42]. In the F3 AD system (insoluble natural cellulose represented by degreased cotton), this genetic profile implies that microbial communities might possess highly efficient adaptive strategies for foraging and surface colonization. We hypothesize that the enriched pathways for chemotactic sensing and flagellar propulsion could facilitate microorganisms to migrate toward undegraded cotton fibers and acquire localized nutrients through direct physical contact. Microscopic visualization could further serve as compelling evidence to corroborate the above inference. Such potential substrate-interaction capabilities might enhance the utilization efficiency of solid cellulose substrates and likely maintained the long-term degradation stability of the F3 reactor.

**Figure 5:**
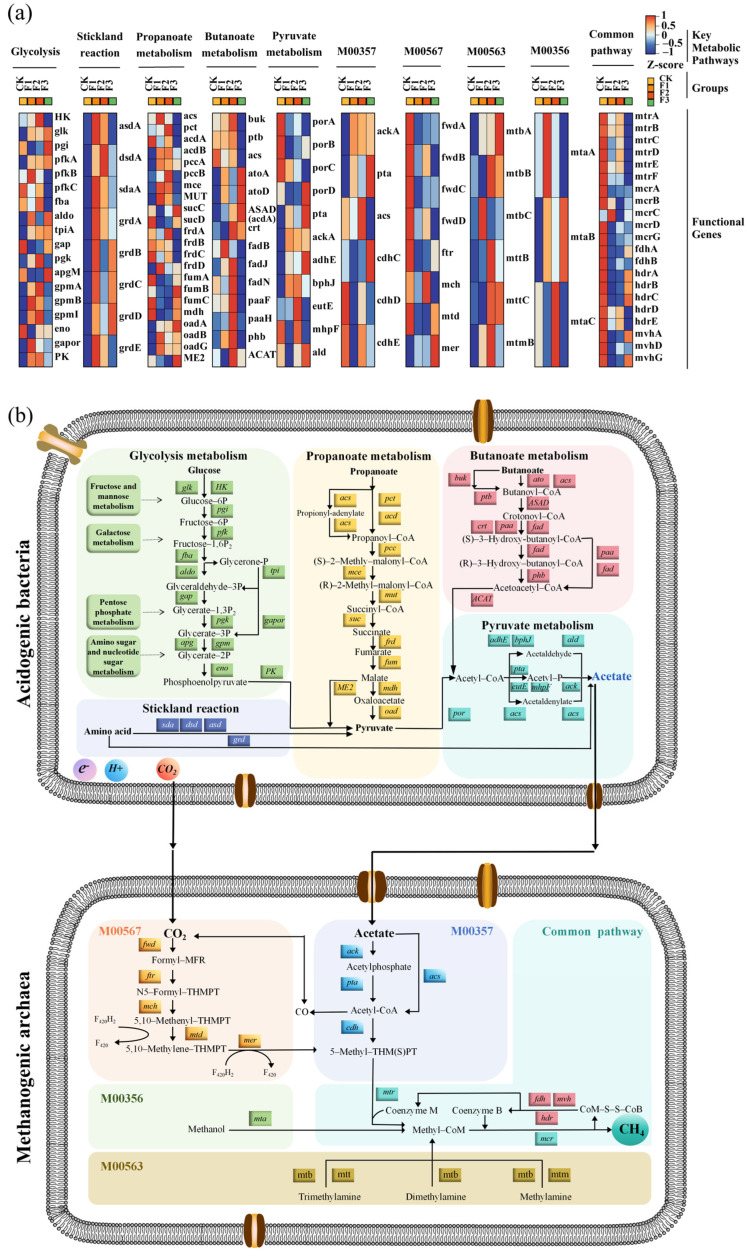
Multiple group heatmap of relative abundances of functional genes (**a**) and key metabolic pathways of acidogenesis and methanogenesis (**b**) in the anaerobic digestion process.

To clarify the potential metabolic mechanisms underlying the natural cellulose anaerobic digestion system, this study reconstructed the predictive metabolic pathways encompassing acidogenesis and methanogenesis (Figure 5). In the upstream metabolic pathways, glycolysis-related genes (e.g., *glk*, *pfkA*, *fba*, and *gap*) were significantly enriched in F1. The continuous and rapid extracellular depolymerization of complex cellulosic carbohydrates supplied abundant monosaccharides, which were subsequently channeled into the glycolytic pathway. The high enrichment of glycolysis-related functional genes was closely correlated with the predominance of Firmicutes in the F1 group. It provided a molecular-level explanation for the excellent cellulose conversion efficiency of approximately 88% observed in this group. Furthermore, genes involved in pyruvate metabolism—specifically the acetate kinase gene (*ackA*) and the phosphate acetyltransferase gene (*pta*), which were critical for the conversion of acetyl-CoA to acetate—exhibited highly elevated abundances in both groups F1 and F2. F1 and F2 exhibited more significant acetic acid production capacity, which could provide sufficient precursor substrates for the subsequent methanogenesis process (Figure 3b). The volatile fatty acid (VFA) profile in all three groups was dominated by acetic acid during the acidification phase, suggesting that fermentation mainly followed the acetic acid-type pathway. Volatile fatty acid (VFA) accumulation (propionate and butyrate) frequently induces thermodynamic stress and prolongs the lag phase in anaerobic digestion. The multiple group heatmap demonstrated that genes encoding propionate and butyrate metabolism (*buk*, *ptb*, *pct*, and *fad*) were significantly more enriched in the F1 and F2 groups than in F3 (Figure 5a). Such elevated genetic potential implied a robust syntrophic oxidation network, potentially allowing F1 and F2 to rapidly oxidize excessive VFAs into acetate and hydrogen, thereby effectively avoiding system acidification [43].

Significant variations in the genetic potential of methanogenic pathways were observed among the treatment groups. In F1 and F2, the genetic capacity for methanogenesis was primarily dominated by the acetoclastic pathway (Module M00357), as evidenced by the high relative abundance of the *ackA* and *acs*. This functional profile was consistent with microbial community structure, which showed the predominance of *Methanothrix* in two groups. However, a distinct shift in metabolic potential occurred in F3. The relative abundance of key genes in the acetoclastic module (M00357) was markedly reduced, while genes involved in the hydrogenotrophic pathway (Module M00567), specifically *ftr*, *mtd*, and *mer*, were significantly enriched. It indicated that the genetic capacity for acetoclastic methanogenesis was constrained in F3, suggesting that the archaeal community might rely more heavily on CO2-reducing methanogenesis. Given that the establishment of hydrogenotrophic syntropy typically required a longer adaptation period, this transition in functional potential provided a plausible molecular-level explanation for the extended lag phase observed in F3. In the CK group (only inoculum), the lack of exogenous carbon sources resulted in a substantially lower abundance of acetoclastic genes compared to the hydrogenotrophic pathway. In addition, the methylotrophic pathway (Module M00563) was identified as the third essential route for methanogenesis. The enrichment of functional genes related to the methylotrophic pathway across three groups suggested that supplementation with natural cellulose substrates not only influenced upstream hydrolysis and acidogenesis but also reshaped the downstream methanogenic potential profiles, thereby driving the functional potential diversification of the anaerobic system.

## 4. Conclusions

This study demonstrated that natural cellulose could be significantly degraded and converted into methane by anaerobic microorganisms. Cellulose is the predominant chemical component in three feedstocks, and degreased cotton fiber possessed the highest crystallinity among the three types of fibers. The cumulative specific methane yields of wood pulp fiber (F1), straw pulp fiber (F2) and degreased cotton fiber (F3) were 373.57 ± 10.70 mL/g VS, 349.15 ± 13.20 mL/g VS and 346.16 ± 1.60 mL/g VS respectively, and the corresponding biodegradability values were 93.97%, 85.95% and 84.32%. Although the T95 time (12 days) was same in F1, F2 and F3, different biogas production patterns were exhibited by the three groups. A higher biomethane production rate was achieved in F1 during AD, whereas a relatively low biogas production intensity was observed in F2. In comparison, F3 was characterized by slow and continuous biogas generation over the fermentation period. Metagenomic analysis indicated that F1 and F2 were dominated by the acetoclastic methanogenesis pathway, while the proportion of the hydrogenotrophic methanogenesis pathway increased in group F3. Meanwhile, the cell motility pathway category (KEGG Level 2) was significantly enriched in the AD system of F3, indicating that the microbial community could form an efficient adaptive mechanism for the AD of cellulose.

## Figures and Tables

**Table 1:** Characteristics of inoculum and feedstocks.

Characteristics	Inoculum	F1	F2	F3
TS (%) ^a^	10.67 ± 0.10	95.86 ± 0.06	96.94 ± 0.08	97.08 ± 0.04
VS (%) ^a^	8.01 ± 0.12	95.77 ± 0.07	94.16 ± 0.06	97.08 ± 0.04
NH_3_-N (mg/L)	621.33 ± 24.07	N.A.	N.A.	N.A.
Lignin (%) ^b^	1.34 ± 0.07	1.76 ± 0.98	4.28 ± 0.67	N.D.
Cellulose (%) ^b^	1.00 ± 0.12	92.89 ± 0.77	84.16 ± 1.01	97.19 ± 0.40
Hemicellulose (%) ^b^	1.30 ± 0.36	5.45 ± 0.41	6.78 ± 0.04	2.68 ± 0.27
C (%) ^b^	38.86 ± 0.24	43.69 ± 0.04	42.04 ± 0.02	44.50 ± 0.01
H (%) ^b^	5.48 ± 0.27	5.84 ± 0.05	6.17 ± 0.05	6.01 ± 0.07
N (%) ^b^	8.74 ± 0.07	N.D.	0.13 ± 0.01	N.D.
O (%) ^b^	42.76 ± 0.38	50.08 ± 0.08	48.66 ± 0.05	49.47 ± 0.05
Water-soluble sulfates (mg/kg)	N.A.	93.97	91.87	N.D.
Water-soluble chlorides (mg/kg)	N.A.	243.8	N.D.	N.D.

^a^ based on wet weight, ^b^ based on dry weight, N.A., Not applicable. N.D., Not detected.

**Table 2:** Kinetic analysis of methane yield.

Fitting Model	Parameters	F1	F2	F3
Modified Gompertz model	*B*_0_ (mL/g VS)	371.01	346.46	347.95
	*R*_max_ (mL/g VS/d)	69.58	53.71	54.64
	*λ* (d)	2.24	2.32	2.72
	R^2^ (%)	99.74%	99.69%	99.95%
Logistic model	*B*_0_ (mL/g VS)	367.62	342.48	344.42
	*R*_max_ (mL/g VS/d)	69.40	53.28	54.27
	*λ* (d)	2.39	2.49	2.94
	R^2^ (%)	99.09	98.94	99.65
Cone model	*B*_0_ (mL/g VS)	375.61	353.26	352.79
	*k* (d^−1^)	0.20	0.18	0.17
	R^2^ (%)	99.86%	99.82%	99.88%
First-order kinetics model	*B*_0_ (mL/g VS)	407.35	393.64	400.06
	*k* (d^−1^)	0.14	0.12	0.11
	R^2^ (%)	92.63%	93.37%	92.05%

## Data Availability

The original contributions presented in this study are included in the article/Supplementary Materials. Further inquiries can be directed to the corresponding author.

## Supplementary Materials

The following supporting information can be downloaded at: https://www.mdpi.com/article/10.3390/bioengineering13060613/s1, Figure S1: Visual appearance and SEM micro-graphs of different natural cellulose fibers: softwood pulp fiber (a,d), straw pulp fiber (b,e), and degreased cotton fiber (c,f); Figure S2: Daily biogas production (a), cumulative biogas production (b) from different anaerobic digestion groups, and the percentage content of CH_4_ (c), CO_2_ (d) in biogas; Figure S3: Curve fitting of Modified Gompertz model (a), Logistic model (b), Cone model (c) and First-order kinetics model (d); Figure S4: Circos plot showing the distribution of dominant microbial communities at the genus level across different groups. In the plot, the left semi-circle represents the four treatment groups (CK, F1, F2, and F3), while the right semi-circle designates the dominant microbial taxa. The inner circle represents the total relative abundance of specific taxa or groups. The outer circle provides a percentage scale for specific quantification. The connecting ribbons indicate the correspondence between the groups and the microbial genera, with the width of each ribbon being directly proportional to the relative abundance of a specific genus within a given group. Different colors are assigned to distinguish individual microbial taxa and their corresponding distribution flows; Figure S5: Bipartite network diagram illustrating the associations between the treatment groups and the top 200 dominant microbial taxa at the genus level. The large nodes with bold text represent the four experimental groups (CK, F1, F2, and F3), while the smaller nodes represent the specific microbial taxa. The connecting edges indicate the presence of a taxon in a respective group, with the thickness of the edgesbeing directly proportional to the absolute abundance intensity of that taxon within the specific group. The varying colors of the taxonomic nodes represent. Initially, the top 200 abundant genera were selected. Subsequently, a filtering threshold was applied during network visualization to retain only edges representing substantial absolute abundance, resulting in a finalized network of the most prominently associated taxa.The five highlighted medium-sized colored nodes are the top 5 genera ranked by absolute abundance, Methanothrix, unclassified_f_Methanobacteriaceae, Methanobacterium, unclassified_p_Chloroflexi, and unclassified_o_Bacteroidales; Figure S6: Absolute abundance of microbial functional pathways annotated at KEGG level 1 (a), level 2 (b) and top 20 level 3 (c) across CK, F1, F2 and F3. Absolute abundance of specific functions related to cell mobility at KEGG pathway level 3 (d). Overall, Metabolism was the dominant functional category across all groups. Within the Cellular Processes category, the absolute abundance of the cell motility pathway was significantly increased in the F3 treatment compared with the other groups.
