# Supplementary material for: Methane Yield, Substrate Conversion, Microbial Community Structure and Metabolic Pathways During Anaerobic Digestion of Natural Cellulosic Biomass
Source: Bioengineering (Basel). 2026 May 25;13(6):613. doi: 10.3390/bioengineering13060613 (PMC13295860; doi:10.3390/bioengineering13060613)
Supplement: Supplementary file 1 [file bioengineering-13-00613-s001.zip › bioengineering-4314133-supplementary.pdf]

Supplementary Materials

Methane Yield, Substrate Conversion, Microbial Community Structure and Metabolic Pathways During Anaerobic Digestion of Natural Cellulosic Biomass

Xiteng Chen, Hairong Yuan and Xiujin Li *.
Department of Environmental Science and Engineering, Beijing University of Chemical Technology, Beijing 100029, China
* Correspondence: xjli@mail.buct.edu.cn

There are six figures in this supplementary materials:

Figure S1. Visual appearance and SEM micro-graphs of different natural cellulose fibers: softwood pulp fiber (a)(d), straw pulp fiber (b)(e), and degreased cotton fiber (c)(f)

Figure S2. Daily biogas production (a), cumulative biogas production (b) from different anaerobic digestion groups, and the percentage content of CH4 (c), CO2 (d) in biogas

Figure S3. Curve fitting of Modified Gompertz model (a), Logistic model (b), Cone model (c), and First-order kinetics model (d)

Figure S4. Circos plot showing the distribution of dominant microbial communities at the genus level across different groups. In the plot, the left semi-circle represents the four treatment groups (CK, F1, F2, and F3), while the right semi-circle designates the dominant microbial taxa. The inner circle represents the total relative abundance of specific taxa or groups. The outer circle provides a percentage scale for specific quantification. The connecting ribbons indicate the correspondence between the groups and the microbial genera, with the width of each ribbon being directly proportional to the relative abundance of a specific genus within a given group. Different colors are assigned to distinguish individual microbial taxa and their corresponding distribution flows

Figure S5. Bipartite network diagram illustrating the associations between the treatment groups and the top 200 dominant microbial taxa at the genus level. The large

nodes with bold text represent the four experimental groups (CK, F1, F2, and F3), while the smaller nodes represent the specific microbial taxa. The connecting edges indicate the presence of a taxon in a respective group, with the thickness of the edgesbeing directly proportional to the absolute abundance intensity of that taxon within the specific group. The varying colors of the taxonomic nodes represent. Initially, the top 200 abundant genera were selected. Subsequently, a filtering threshold was applied during network visualization to retain only edges representing substantial absolute abundance, resulting in a finalized network of the most prominently associated taxa.The five highlighted medium-sized colored nodes are the top 5 genera ranked by absolute abundance, *Methanothrix, unclassified_f_Methanobacteriaceae, Methanobacterium, unclassified_p_Chloroflexi*, and *unclassified_o_Bacteroidales*

Figure S6. Absolute abundance of microbial functional pathways annotated at KEGG level 1 (a), level 2 (b) and top 20 level 3 (c) across CK, F1, F2 and F3. Absolute abundance of specific functions related to cell mobility at KEGG pathway level 3 (d). Overall, Metabolism was the dominant functional category across all groups. Within the Cellular Processes category, the absolute abundance of the cell motility pathway was significantly increased in the F3 treatment compared with the other groups

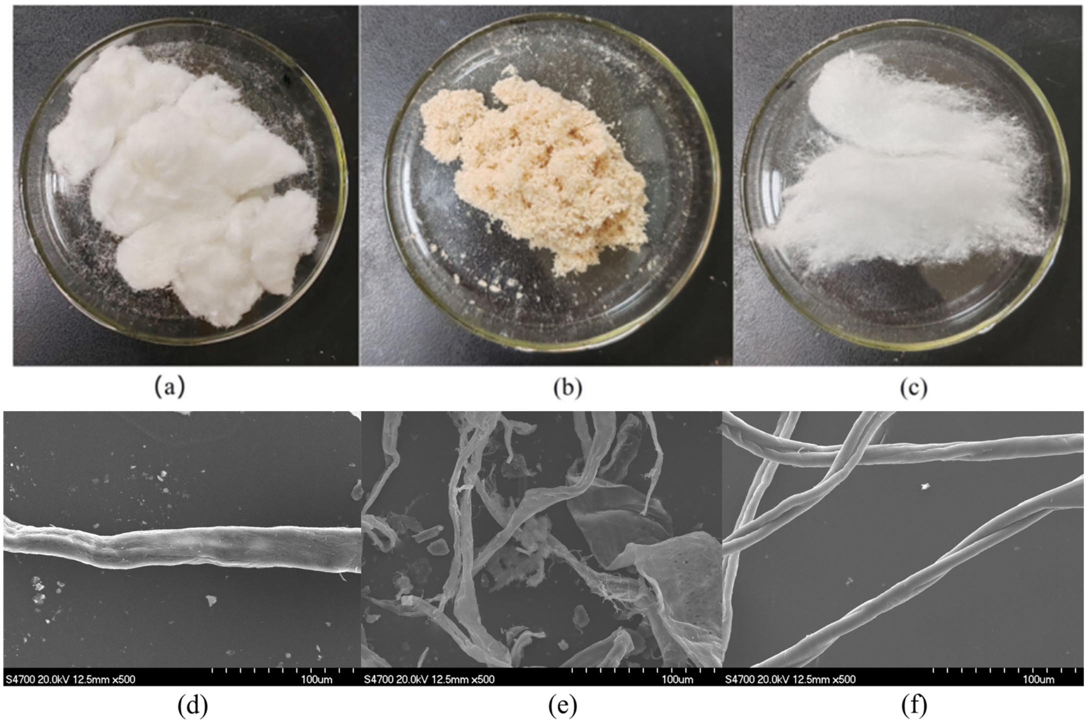

Figure S1. Visual appearance and SEM micro-graphs of different natural cellulose fibers: softwood pulp fiber (a)(d), straw pulp fiber (b)(e), and degreased cotton fiber (c)(f)

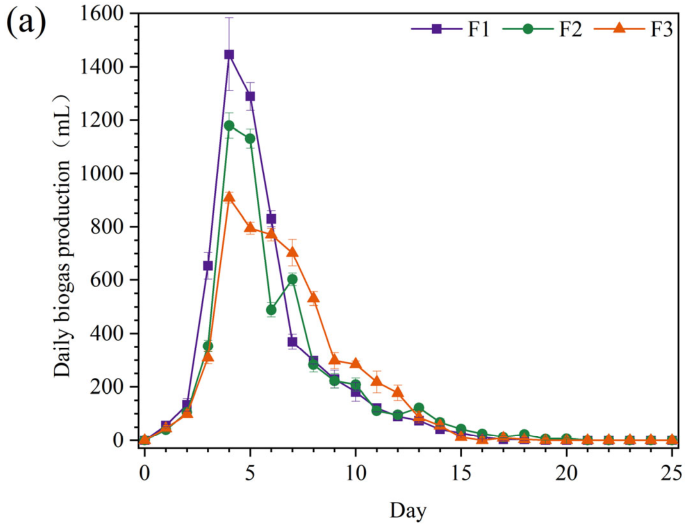

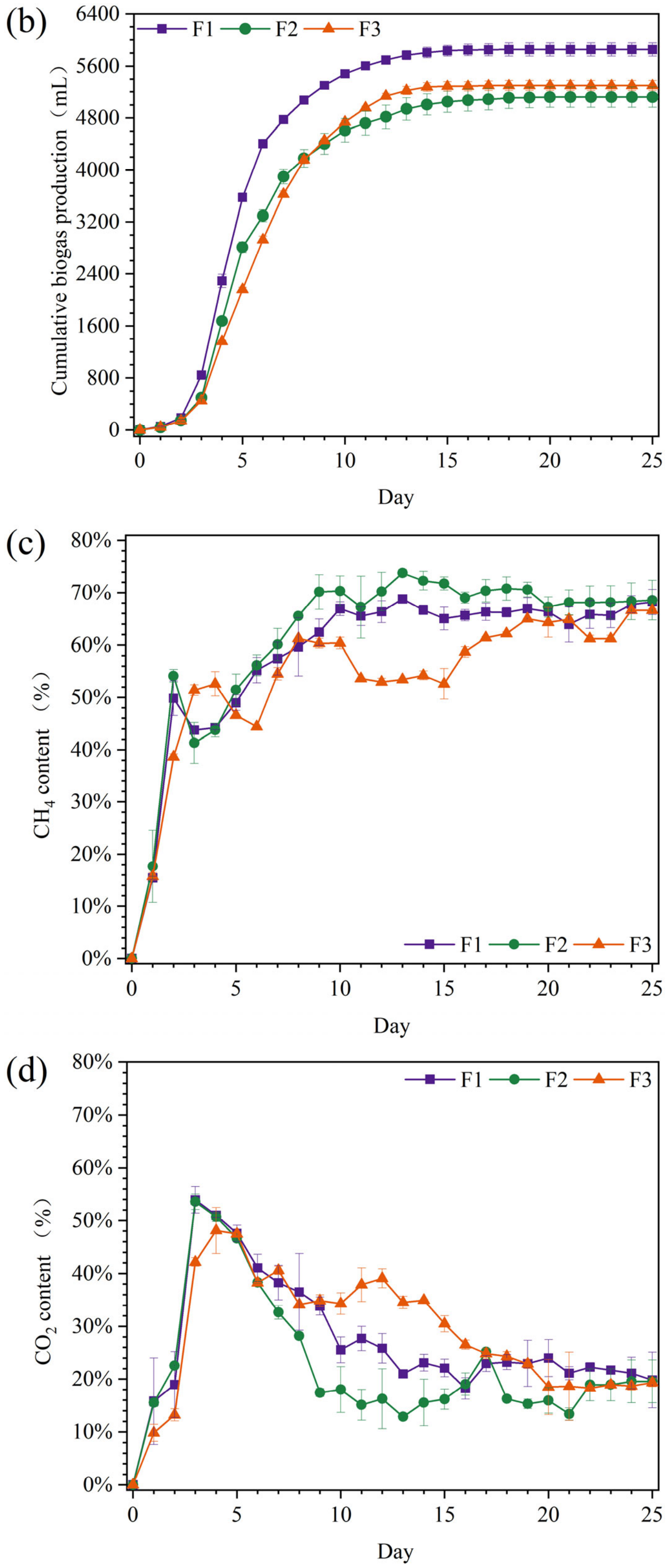

Figure S2. Daily biogas production (a), cumulative biogas production (b) from different anaerobic digestion groups, and the percentage content of CH_4 (c), CO_2 (d) in biogas

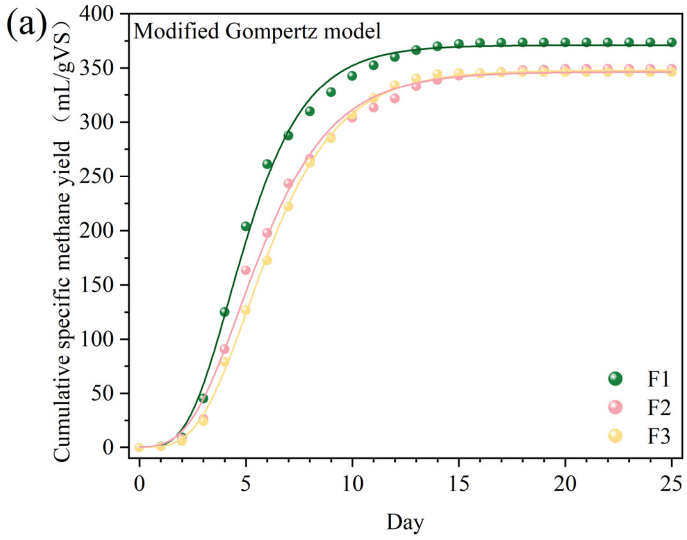
(a)
Modified Gompertz model
Cumulative specific methane yield （mL/gVS）
Day
F1
F2
F3
0
50
100
150
200
250
300
350
400
0
5
10
15
20
25

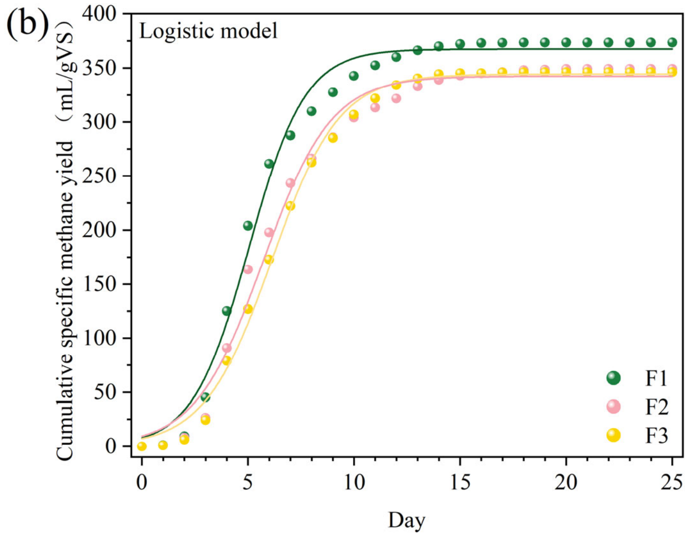
(b)
Logistic model
Cumulative specific methane yield （mL/gVS）
Day
F1
F2
F3
0
50
100
150
200
250
300
350
400
0
5
10
15
20
25

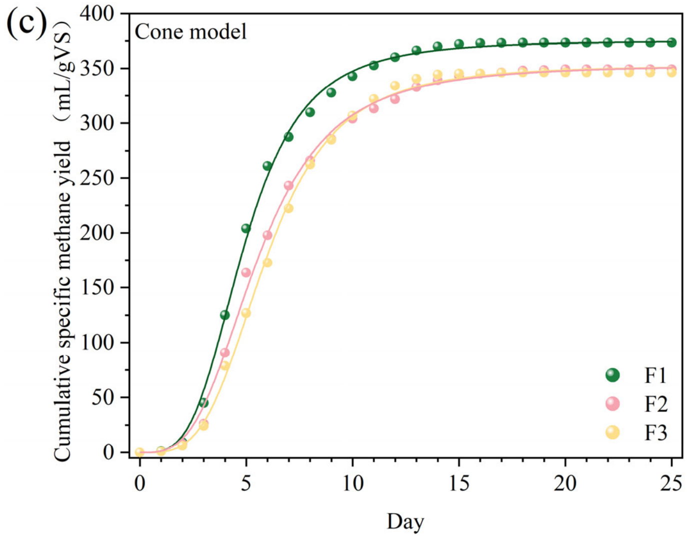
(c)
Cone model
Cumulative specific methane yield （mL/gVS）
Day
F1
F2
F3
0
50
100
150
200
250
300
350
400
0
5
10
15
20
25

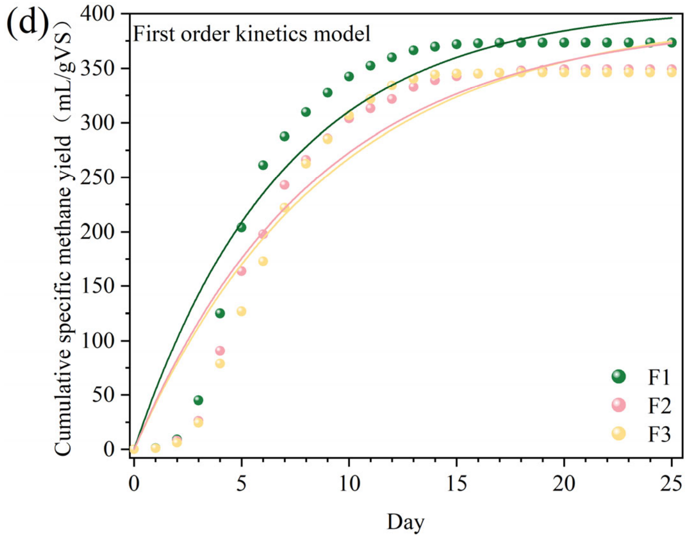

Figure S3. Curve fitting of Modified Gompertz model (a), Logistic model (b), Cone model (c) and First-order kinetics model (d)

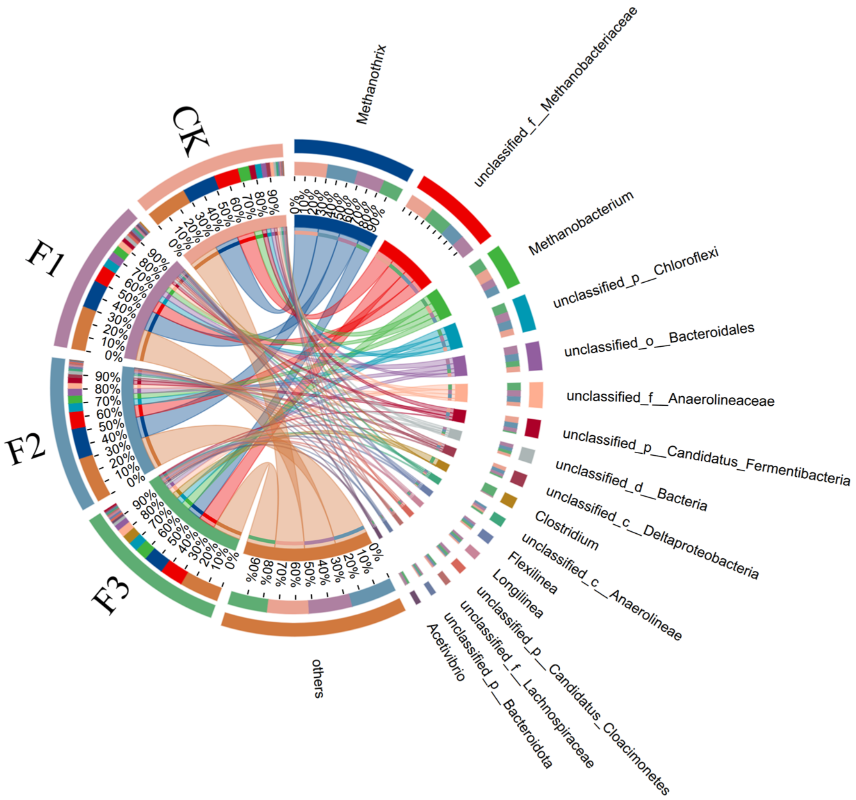

Figure S4. Circos plot showing the distribution of dominant microbial communities at the genus level across different groups. In the plot, the left semi-circle represents the four treatment groups (CK, F1, F2, and F3), while the right semi-circle designates the dominant microbial taxa. The inner circle represents the total relative abundance of specific taxa or groups. The outer circle provides a percentage scale for specific quantification. The connecting ribbons indicate the correspondence between the groups and the microbial genera, with the width of each ribbon being directly proportional to the relative abundance of a specific genus within a given group. Different colors are assigned to distinguish individual microbial taxa and their corresponding distribution flows

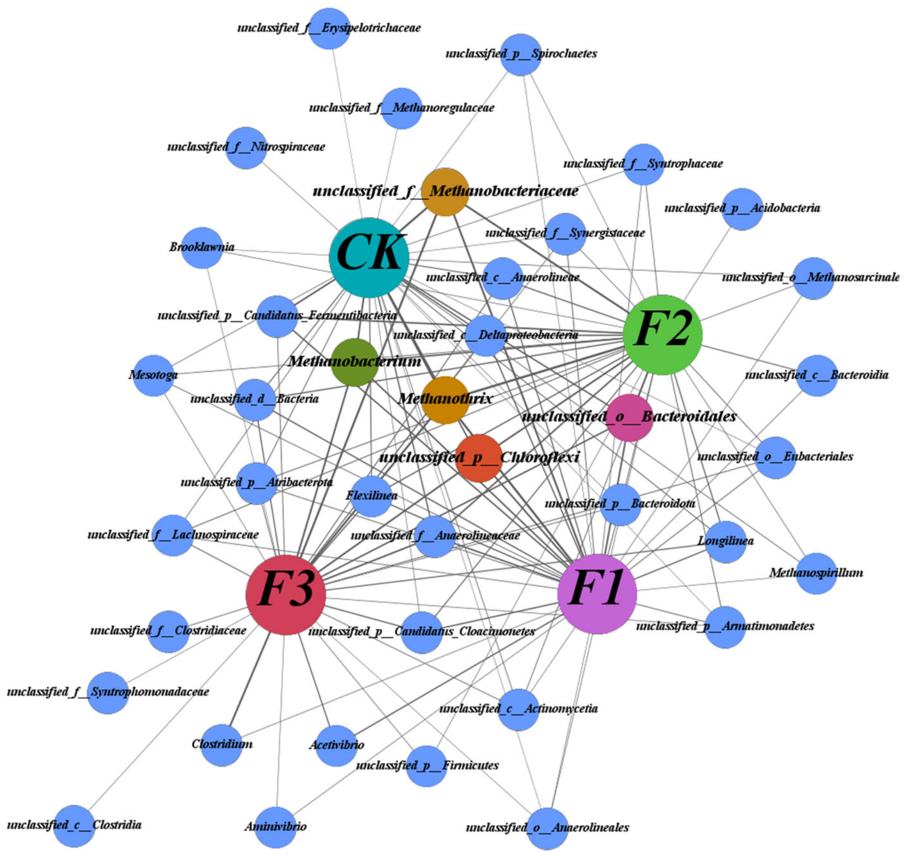

Figure S5. Bipartite network diagram illustrating the associations between the treatment groups and the top 200 dominant microbial taxa at the genus level. The large nodes with bold text represent the four experimental groups (CK, F1, F2, and F3), while the smaller nodes represent the specific microbial taxa. The connecting edges indicate the presence of a taxon in a respective group, with the thickness of the edgesbeing directly proportional to the absolute abundance intensity of that taxon within the specific group. The varying colors of the taxonomic nodes represent. Initially, the top 200 abundant genera were selected. Subsequently, a filtering threshold was applied during network visualization to retain only edges representing substantial absolute abundance, resulting in a finalized network of the most prominently associated taxa.The five highlighted medium-sized colored nodes are the top 5 genera ranked by absolute abundance, *Methanothrix*, *unclassified_f_Methanobacteriaceae*, *Methanobacterium*, *unclassified_p_Chloroflexi*, and *unclassified_o_Bacteroidales*

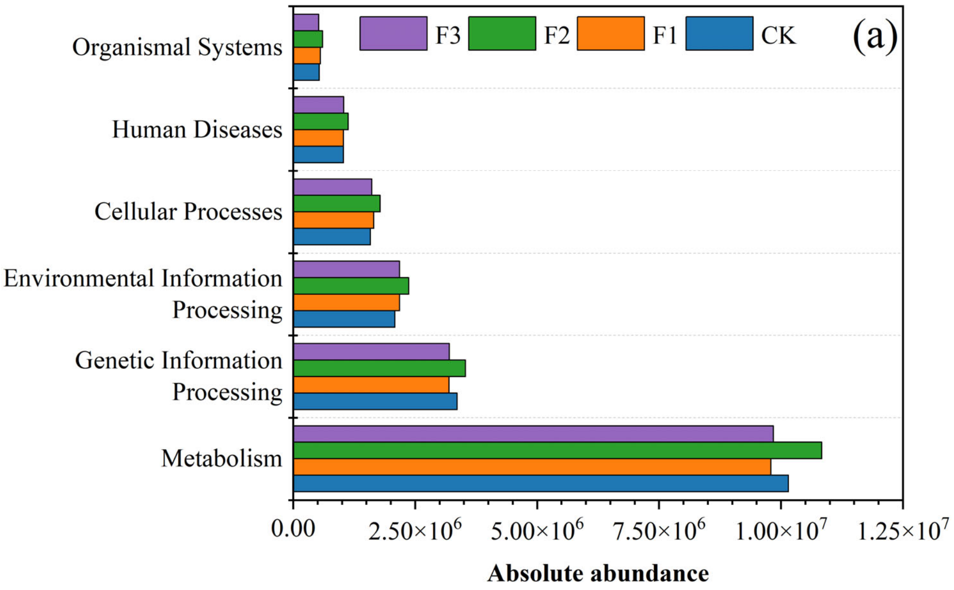
F3
F2
F1
CK
(a)
Organismal Systems
Human Diseases
Cellular Processes
Environmental Information Processing
Genetic Information Processing
Metabolism
0.00
2.50×10^6
5.00×10^6
7.50×10^6
1.00×10^7
1.25×10^7
Absolute abundance

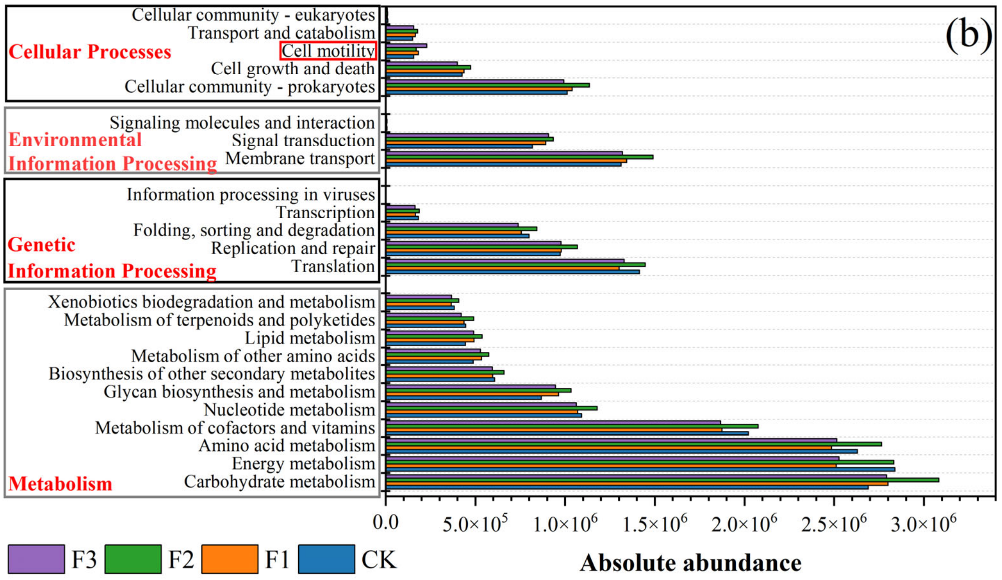
(b)
Cellular Processes
Cellular community - eukaryotes
Transport and catabolism
Cell motility
Cell growth and death
Cellular community - prokaryotes
Environmental Information Processing
Signaling molecules and interaction
Signal transduction
Membrane transport
Genetic Information Processing
Information processing in viruses
Transcription
Folding, sorting and degradation
Replication and repair
Translation
Metabolism
Xenobiotics biodegradation and metabolism
Metabolism of terpenoids and polyketides
Lipid metabolism
Metabolism of other amino acids
Biosynthesis of other secondary metabolites
Glycan biosynthesis and metabolism
Nucleotide metabolism
Metabolism of cofactors and vitamins
Amino acid metabolism
Energy metabolism
Carbohydrate metabolism
0.0
5.0×10^5
1.0×10^6
1.5×10^6
2.0×10^6
2.5×10^6
3.0×10^6
F3
F2
F1
CK
Absolute abundance

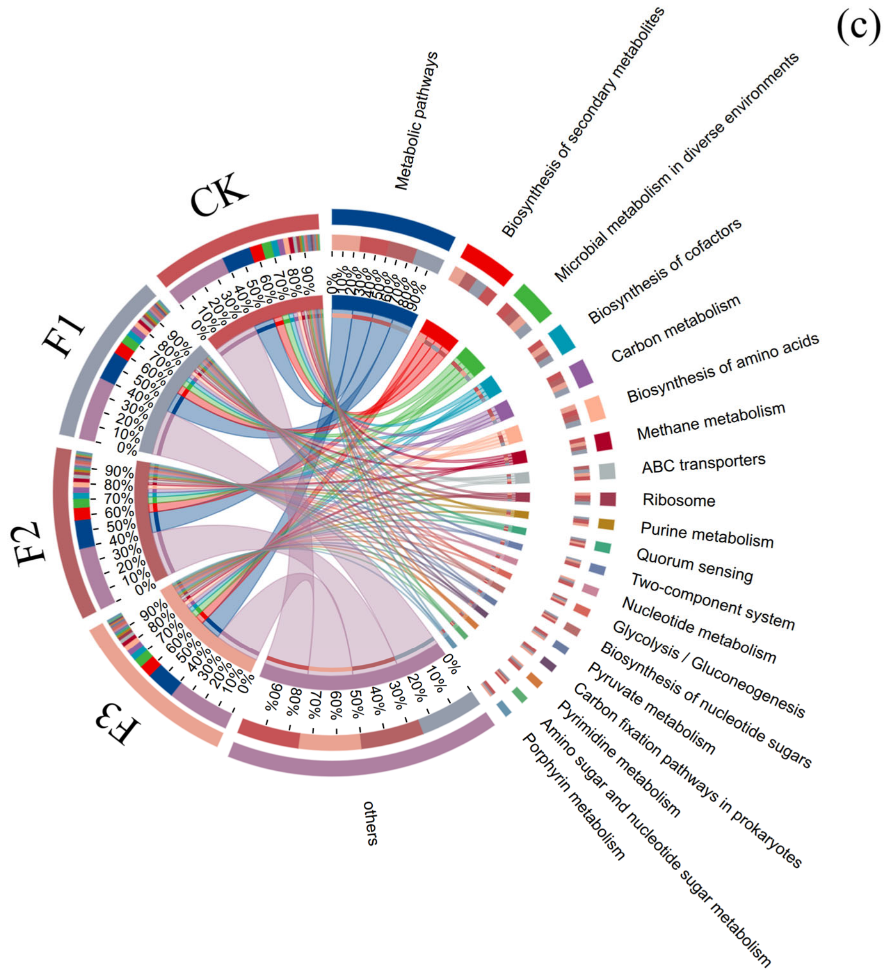

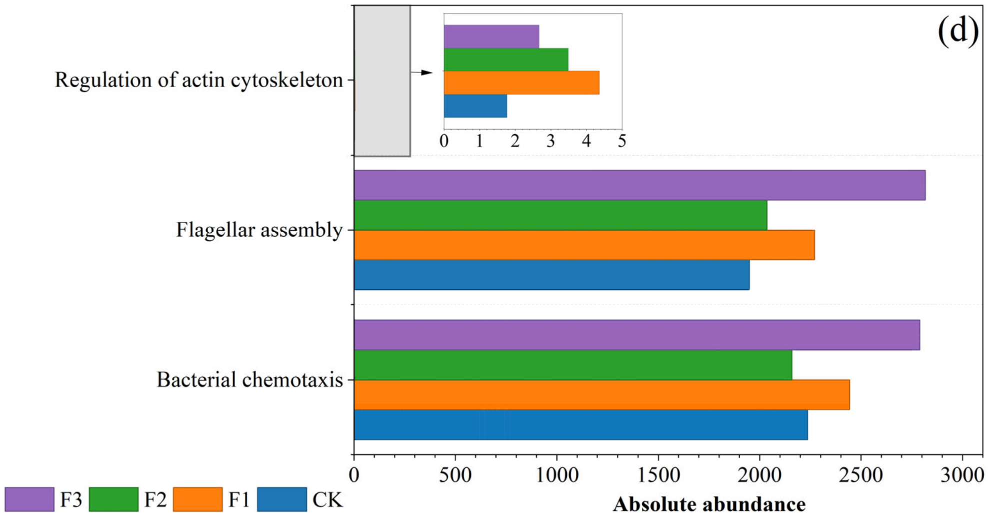

Figure S6. Absolute abundance of microbial functional pathways annotated at KEGG level 1 (a), level 2 (b) and top 20 level 3 (c) across CK, F1, F2 and F3. Absolute abundance of specific functions related to cell mobility at KEGG pathway level 3 (d). Overall, Metabolism was the dominant functional category across all groups. Within the Cellular Processes category, the absolute abundance of the cell motility pathway was significantly increased in the F3 treatment compared with the other groups
